# Anxiety, Depression, and Colorectal Cancer Survival: Results from Two Prospective Cohorts

**DOI:** 10.3390/jcm9103174

**Published:** 2020-09-30

**Authors:** Claudia Trudel-Fitzgerald, Shelley S. Tworoger, Xuehong Zhang, Edward L. Giovannucci, Jeffrey A. Meyerhardt, Laura D. Kubzansky

**Affiliations:** 1 Department of Social and Behavioral Sciences, Harvard T.H. Chan School of Public Health, Boston, MA 02115, USA; lkubzans@hsph.harvard.edu; 2 Division of Population Science, Moffitt Cancer Center, Tampa, FL 33612, USA; Shelley.Tworoger@moffitt.org; 3 Department of Epidemiology, Harvard T.H. Chan School of Public Health, Boston, MA 02115, USA; egiovann@hsph.harvard.edu; 4 Channing Division of Network Medicine, Department of Medicine Brigham and Women’s Hospital and Harvard Medical School, Boston, MA 02115, USA; poxue@channing.harvard.edu; 5 Department of Medical Oncology, Dana-Farber Cancer Institute and Harvard Medical School, Boston, MA 02115, USA; Jeffrey_Meyerhardt@dfci.harvard.edu; 6 Department of Nutrition, Harvard T.H. Chan School of Public Health, Boston, MA 02115, USA

**Keywords:** anxiety, anxiolytics, antidepressants, cancer, colorectal cancer, depression, health behaviors, mortality, psychological distress, survival

## Abstract

Given the unalterable nature of most risk factors for colorectal cancer (CRC) survival (e.g., disease stage), identifying modifiable determinants is critical. We investigated whether anxiety and depression were related to CRC survival using data from the Nurses’ Health Study (NHS) and Health Professional Follow-up Study (HPFS). Participants who received a CRC diagnosis and provided information about anxiety (n_NHS_ = 335; n_HPFS_ = 232) and depression (n_NHS_ = 893; n_HPFS_ = 272) within 4 years of diagnosis were included. Cox regression models estimated hazard ratios (HR) and 95% confidence intervals (CI) of overall mortality, while controlling for covariates (sociodemographics, cancer characteristics, and lifestyle factors). Pooled risk estimates were derived from fixed effects meta-analyses of the cohorts. Among 1732 CRC patients, 814 deaths occurred during the 28-year follow-up. Each 1 standard deviation increase in anxiety or depression symptoms was associated with a similar 16% higher mortality risk (anxiety: 95% CI = 1.05–1.29; depression: 95% CI = 1.07–1.26). Comparable results were observed across all sensitivity analyses (introducing a 1-year lag, restricting to CRC-related mortality, considering potential behavioral pathways) and stratified models (cancer stage, sex). Our findings suggest greater anxiety and depression symptoms can not only impede adherence to healthy habits and reduce quality of life in cancer patients but could also be a marker for accelerated CRC progression.

## 1. Introduction

Colorectal cancer (CRC) is one of the most common cancers worldwide, with cases occurring mainly in adults 50 years or older [1,2]. Despite higher numbers of long-term CRC survivors due to advances in detection and treatment, nearly half are at high risk of mortality in the 10 years following diagnosis [1]. Cancer characteristics, including advanced stage and proximal tumors, are associated with reduced survival [1,3]. As these characteristics are unalterable, identifying modifiable determinants of CRC survival, particularly in midlife adults, is imperative. Anxiety and depression, which are common mental disorders, are treatable. Although substantial evidence has linked anxiety- and depression-related symptoms/ disorders with higher cardiovascular disease and mortality risk [4,5,6], cancer-related findings are limited, inconsistent, and still debated [6,7,8,9,10,11,12,13].

Because cancer is highly heterogeneous [2,14,15], studies combining different sites (e.g., skin, lung) may mask relationships with anxiety/depression [10,15,16,17], contributing to inconsistent findings. When considering mortality in CRC patients specifically, findings regarding the role of anxiety and depression are scarce but suggestive. Notably, in one study greater self-reported symptoms of depression, but not anxiety, assessed post-diagnosis in 421 patients with metastatic CRC were associated with increased mortality risk over a median follow-up of 20 months (hazard ratio (HR) = 2.04; 95% confidence intervals (CI) = 1.52–2.70) [18]. Likewise, results from another study based on post-diagnosis psychiatric interviews indicated CRC patients with versus without a common mental disorder were more likely to die of cancer (HR = 1.35; 95% CI = 1.20–1.53) over 3 years on average (*n* = 28,007; mostly localized stage) [16]. Prior studies using emotional functioning scores from quality of life scales showed both positive [19,20] and null [21] findings, potentially because these assess a more general construct than measures of anxiety and depression.

Other methodological limitations may contribute to mixed evidence. Most studies do not evaluate whether psychotropics, frequently prescribed to cancer patients [22], affect study findings [23]. Because individuals using antidepressants/anxiolytics may be more likely to report fewer anxiety- or depression-related symptoms, and common mental disorders are often measured using self-report symptoms, failure to account for use of psychotropics may lead to exposure misclassification. Additionally, because participants must have survived until the psychological assessment, analytic samples can include healthier individuals who may have better initial mental/physical states, resulting in potential selection bias [24].

Investigations addressing these conceptual and methodological issues may help to clarify the role of common mental disorders in cancer survival. Therefore, the current study leverages existing data from two long-running prospective cohorts to investigate whether anxiety and depression, assessed comprehensively by self-reported symptom scales, physician diagnosis, and psychotropics use after cancer diagnosis, were related to mortality risk over up to 28 years among CRC patients. To do this, we harmonized data across numerous measures and analytic samples, in order to optimize both sample size available for analyses and also utilize the most rigorous study design possible. Statistical analyses included inverse probability weighting to account for potential selection bias. We hypothesized that higher anxiety and depressive symptoms, characterized either as self-reported symptoms only or as a combination of symptoms, physician diagnosis, and psychotropics use, would be associated with a greater mortality risk over follow-up. 

## 2. Methods

### 2.1. Participants

The Nurses’ Health Study (NHS) is an ongoing cohort comprised of 121,700 U.S. female nurses, ages 30–55 years in 1976, and the Health Professional Follow-up Study (HPFS) began in 1986 among 51,529 male health professionals who were 40–75 years. Participants from these complementary cohorts have completed biennial questionnaires on lifestyle, medical history and newly diagnosed medical conditions, with sustained high response rates [25,26,27].

The present study includes NHS/HPFS individuals diagnosed with CRC, who did not report having prior cancer except non-melanoma skin cancer, and who provided data for at least two indicators of anxiety or depression (i.e., self-reported scales, medication use, and/or physician diagnosis) within 4 years following CRC diagnosis. Following similar work [28,29], this 4-year timeframe was selected to both ensure sufficiently large sample sizes for conducting survival analyses and also optimize the likelihood that anxiety/depression symptoms were related to cancer. This post-diagnosis psychological assessment represents the study baseline for each participant. As detailed in Text S1, psychological symptoms were often, but not always, assessed before CRC diagnosis in the larger NHS/HPFS cohorts, and only a small subset of our analytic samples had completed such measures. Moreover, when the measures were available they were obtained from 8 to 12 years before the post-diagnosis measures; this lag renders such measures less informative given prior evidence suggesting anxiety or depression are generally stable when comparing their levels before versus many years after receiving a diagnosis of a major medical condition, including cancer [30,31]. As a result, we did not include information about pre-diagnosis distress in the current analyses.

Upon participants’ consent, a physician blinded to study hypotheses reviewed medical records to confirm CRC diagnosis and extract relevant cancer characteristics (e.g., stage). To be eligible for the current study, participants were required to have data on relevant variables, including psychological, sociodemographics, and lifestyle factors at the analytic baseline, resulting in samples of N_anxiety_ = 567; N_depression_ = 1165 for the meta-analyses (see flowchart for sample eligibility and inclusion, Figure S1). Because depression was assessed more frequently than anxiety, more individuals were available for that analysis. Participants who did versus did not complete a psychological assessment had higher prevalence of cardiometabolic disease, proximal tumor, and advanced CRC but did not differ meaningfully on other factors (Table S1). The study protocol was approved by the institutional review boards of the Brigham and Women’s Hospital, Harvard T.H. Chan School of Public Health, and those of participating registries (IRB Protocol Numbers 1999-P-011114 and 1999P011117).

### 2.2. Measures

#### 2.2.1. Anxiety and Depression 

To capture psychological symptoms comprehensively and lower the likelihood of misclassification, various indicators were considered: validated symptom scales, physician-diagnosed depression (anxiety was not queried), and psychotropic medication use. Figure S2 depicts the assessments of anxiety- and depression-related indicators over time within NHS and HPFS, respectively. 

##### Self-Reported Anxiety/Depression Symptom Scales

Details about the various selected scales, including score ranges, clinical cutpoints, and temporal stability are provided in Text S1. In brief, for each scale, a continuous and a dichotomized (according to established clinical cut-points) score was considered. Anxiety symptoms were assessed using the 8-item Crown-Crisp Index (CCI) [32] in 2004 in NHS, and in 1988 and 2000 in HPFS. In both cohorts, the 7-item Generalized Anxiety Disorder (GAD-7) scale [33] was administered in 2012. Scores from these self-reported scales are moderately correlated across an 8- to 12-year interval within the larger cohort (e.g., in NHS: CCI_2004_ with GAD-7_2012,_ *r* = 0.31, *p* < 0.0001; in HPFS: CCI_2000_ with GAD-7_2012,_ *r* = 0.25, *p* < 0.0001). Depression symptoms were measured with three instruments within NHS: the 5-item Mental Health Index (MHI-5) from the SF-36 Survey in 1996 and 2000 [34], the 10-item Center for Epidemiologic Studies-Depression Scale (CES-D) in 2004 [35], and the 15-item Geriatric Depression Scale-Short Form (GDS-SF) in 2008 and 2012 [36]. Within HPFS, depressive symptoms were evaluated using the GDS-SF in 2008 and 2012 and using a 1-item screener for depressive symptoms in 2004 and 2008 [37]. In NHS, correlations between scores from these scales are substantial across a 4-year interval (e.g., MHI-5_2000_ with CES-D_2004_, *r* = 0.50, *p* < 0.0001; CES-D_2004_ with GDS-SF_2008_, *r* = 0.50, *p* < 0.0001). In a more detailed investigation of their comparability among NHS women, scores from these three scales were also found to be highly consistent based on the equipercentile equating method [38]. 

##### Self-Reported Clinically-Diagnosed Depression and Psychotropic Medication

Within NHS only, women reported whether a physician had diagnosed depression (yes/no), starting in 2000 on biennial questionnaires. Starting in 1996 on biennial NHS/HPFS questionnaires, participants reported whether they regularly used antidepressants and anxiolytics/tranquilizers within the last 2 years, respectively. We used information on physician-diagnosed depression and medication at time assessments coinciding with symptom scales (Figure S2). In the NHS cohort, physician-diagnosed depression is correlated with both scores of the self-reported depression scales (e.g., CES-D_2004_ with physician-diagnosed_2004,_ point-biserial correlation *r*_pb_ = 0.26, *p* < 0.0001; GDS_2008_ with physician-diagnosed_2008,_ *r*_pb_ = 0.25, *p* < 0.0001) and regular use of antidepressant (e.g., antidepressant use_2004_ with physician diagnosis_2004,_ coefficient Phi *r*_φ_ = 0.46; antidepressant use_2008_ with physician diagnosis_2008,_ *r*_φ_ = 0.47).

To capture continuous anxiety/depression symptoms, total raw scores obtained on symptom scales at each assessment were standardized (z-score metric against sample-specific mean (*M*), standard deviation (*SD*)). We considered imputing symptom scores for those taking psychotropic medication (see details in Text S2); because results when using imputed versus raw scores were similar, for interpretability we used raw scores for all analyses reported. To capture clinical versus non-clinical symptom levels (categorical symptoms), a dichotomized anxiety/depression score was created based on a Boolean OR operator approach [6,39] that leveraged all available information. “Clinical depression” was defined as reporting either (1) depressive symptoms reaching clinical significance (using scales cutpoints); (2) physician-diagnosed depression; or 3) antidepressant use. Likewise, “clinical anxiety” was defined as a score reaching the anxiety scale cutpoint or anxiolytics use. 

#### 2.2.2. Covariates

Cancer characteristics, sociodemographics, health status, and behaviors, available in both cohorts at each analytic baseline (unless specified), were included as covariates following prior evidence [10,16,17,24,40]. Cancer characteristics included age at diagnosis, year of diagnosis, time between diagnosis and analytic baseline, stage, and tumor location. Sociodemographics included age, education level (NHS only, in 1992), census track income (NHS only), occupation (HPFS only, in 1986) and marital status. Health status included prevalent cardiometabolic disease. Health-related behaviors encompassed physical activity, diet quality, smoking, alcohol consumption, and body mass index (BMI), which were aggregated into a lifestyle index following prior studies and cancer guidelines (details in Text S3). 

#### 2.2.3. Deaths Ascertainment

Mortality data was collected from state vital records and the National Death Index and supplemented by reports from family members and postal authorities, leading to 98% mortality follow-up [41]. Physicians blind to study hypotheses ascertained mortality cause from death certificates, supplemented by medical records. Underlying cause of death was assigned according to the International Classification of Diseases, Eighth Revision. Deaths were identified through 1 June 2016. CRC-specific mortality risk represented about a third of all deaths.

### 2.3. Statistical Analysis

Analyses were conducted using SAS^®^ v. 9.4 (SAS Institute, Cary, NC, USA) with a two-sided 0.05 *p*-value. Covariate distribution across anxiety/depression levels was age-standardized, and statistical differences were determined using age-adjusted analyses of variance (ANOVA) and logistic regressions. 

#### 2.3.1. Main Analyses 

Primary analyses include meta-analyzed estimates from each cohort using fixed effects [42]; findings by cohort are reported as additional analyses. Cox regression models assessed the HR and 95% CI of mortality over follow-up (until 2016). For each participant, follow-up starts at the completion of their respective baseline psychological assessment. We used the Schoenfeld residuals to verify the proportional hazards assumptions of the Cox regression models, which suggested no violation of the assumptions. 

Exposure-outcome associations were examined in three sets of models within each cohort with anxiety and depression always considered in separate models/analytic samples. A first model investigated the role of anxiety or depression on mortality risk and controlled for cancer characteristics; as stage data was missing/unspecified for 5–19% of participants across analytic samples, a missing indicator was added. A second model further adjusted for baseline sociodemographics and cardiometabolic diseases. A third, fully-adjusted model further included baseline lifestyle score. N’s for each meta-analysis varied based on available data (see Text S4). Unadjusted Kaplan–Meier curves were also implemented to depict the relation of dichotomized anxiety/depression with overall mortality over follow-up.

#### 2.3.2. Additional Analyses 

In stratified analyses by cohort (i.e., by sex), we used the Cochrane Q statistic to determine heterogeneity in the associations. To examine whether the distress-mortality risk association differed by cancer stage, primary models described above (1) were evaluated among participants with advanced and non-advanced stage separately, and (2) included an interaction term (stage (advanced versus non-advanced) x continuous anxiety/depression symptoms) to evaluate whether associations across cancer stage strata were significantly different. Moreover, we conducted three sensitivity analyses: (1) examining CRC-specific mortality risk; (2) introducing a 1-year lag (i.e., excluding participants who died within the first year) to the primary models to reduce likelihood of reverse causation, whereby underlying carcinogenesis processes would influence the experience or the reporting of anxiety and depression symptoms; (3) in fully-adjusted models that include baseline lifestyle, further adjusting for lifestyle assessed 4 years post-baseline, to allow optimal temporality for evaluating whether behavioral factors might lie on the pathway between anxiety/depression and mortality (in a subset of participants; see Text S4). 

#### 2.3.3. Inverse Probability Weighting

About two-thirds of CRC patients in the larger cohorts completed an anxiety or depression assessment within 4 years of diagnosis (see Table S1). Because unhealthy individuals might be more likely to stop participating or die before this analytic baseline, potential selection and immortal time biases may be present (i.e., included participants differ from excluded ones). Consequently, we used person-specific inverse probability weights to address these issues [24,43]. First, we modeled the probability of completing the baseline anxiety/depression assessment based on time-invariant covariates among CRC patients; then we created a weight corresponding to the inverse probability of having completed the psychological baseline measure and included it in all models. 

## 3. Results

### 3.1. Baseline Characteristics 

Table 1 and Table 2 present the distribution of covariates by baseline anxiety and depression levels separately for NHS (*n* = 1228) and HPFS (*n* = 504). Most participants were married/partnered, and had a non-advanced cancer. Average time between CRC diagnosis and analytic baseline was 2 years. Clinical levels of anxiety and depression were evident in 29.0% and 23.2% of women; rates were lower in men with 22.0% and 17.3%, respectively. Participants with clinical anxiety or depression generally had poorer cardiometabolic health and behaviors; no other consistent differences were evident across groups.

### 3.2. Associations of Anxiety and Depression with Overall Mortality Risk

**Figure 1:**
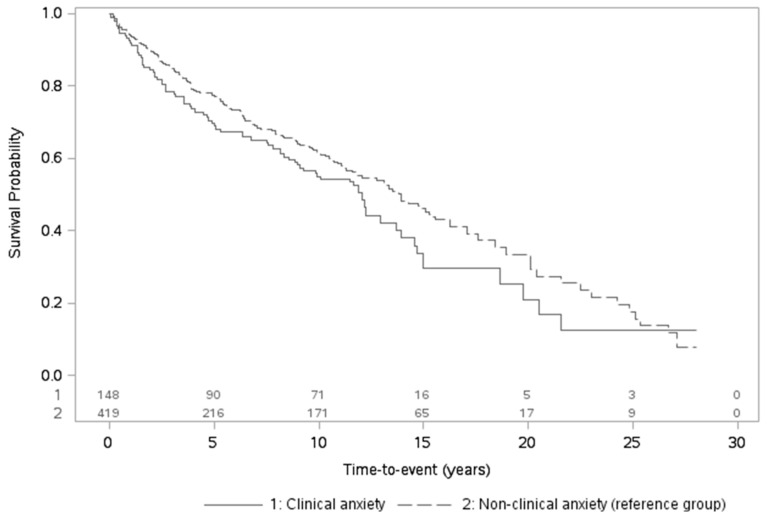
Unadjusted Kaplan-Meier curves for overall mortality in relation to clinical versus non-clinical anxiety levels (including number of participants at risk over time).

**Figure 2:**
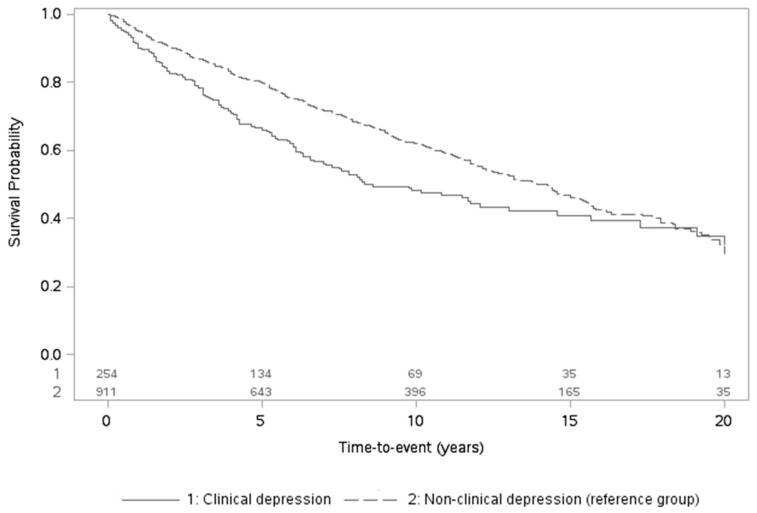
Unadjusted Kaplan-Meier curves for overall mortality in relation to clinical versus non-clinical depression levels (including number of participants at risk over time).

There were 270 (NHS = 122/HPFS = 148) and 544 (NHS = 416/HPFS = 128) deaths in the anxiety and depression analytic samples over 28.0- and 20.0-year follow-up periods, respectively. After meta-analyzing results across cohorts, anxiety and depression were significantly related to elevated mortality risk (Table 3; Figure 1 and Figure 2). Considering continuous exposures in the two analytic samples, each 1-SD rise in anxiety or depression symptoms was associated with 17% and 20% greater mortality risk, respectively, after controlling for cancer characteristics. Further adjusting for sociodemographics and cardiometabolic diseases barely attenuated the estimates (HR_anxiety_ = 1.17, 95% CI = 1.05–1.30; HR_depression_ = 1.17, 95% CI = 1.08–1.27). Associations remained nearly identical after including baseline lifestyle (fully-adjusted HR_anxiety_ = 1.16, 95% CI = 1.05–1.29; HR_depression_ = 1.16, 95% CI = 1.07–1.26). Considering dichotomized exposures, mortality risk was also elevated in patients with versus without clinically relevant levels of anxiety or depression: while CIs became somewhat wider for anxiety, estimates became stronger for depression (fully-adjusted HR_anxiety_ = 1.17, 95% CI = 0.92–1.50; HR_depression_ = 1.28, 95% CI = 1.06–1.56). 

### 3.3. Additional Analyses

The Q statistic, assessing heterogeneity in the associations of anxiety and depression with survival by sex, was not statistically significant (Table 3), except in models considering continuous depression. Specifically, for each 1-SD increase in depression men had approximately 1.5 times greater mortality risk compared to women, with non-overlapping CIs (Table 4). However, only 46 men died in these models. Models stratified by cancer stage revealed similar estimates between non-advanced and advanced tumors (e.g., fully-adjusted models with continuous anxiety: HR_non-advanced_ = 1.18, 95% CI = 1.03–1.36; HR_advanced_ = 1.15, 95% CI = 0.92–1.45; interaction terms *p* ≥ 0.10). 

Across all sensitivity analyses, fully-adjusted estimates were comparably elevated relative to the main estimates. Notably, higher continuous anxiety and depression symptoms were related to a greater CRC-specific mortality risk (e.g., 1-SD increase: HR_anxiety_ = 1.13, 95% CI = 0.93–1.37; HR_depression_ = 1.17, 95% CI = 1.01–1.34). Similarly, in models with a 1-year lag anxiety and depression were associated with increased overall mortality risk (e.g., 1-SD increase: HR_anxiety_ = 1.16, 95% CI = 1.01–1.32; HR_depression_ = 1.21, 95% CI = 1.09–1.34). There was no evidence that lifestyle might lie on the pathway in the relation of either anxiety or depression with mortality risk as HRs and CIs remained nearly identical in the subset of participants who had available lifestyle data at baseline and 4 years later (Table S2; e.g., per 1-SD increase in anxiety: HR_fully-adjusted model with baseline lifestyle_ = 1.20, 95% CI = 1.06–1.35; HR_fully-adjusted model with lifestyle at baseline and 4 years later_ = 1.20, 95% CI = 1.07–1.35). 

## 4. Discussion

This prospective study examined whether anxiety and depression, defined by self-reported symptoms, physician diagnosis, and psychotropic medication, were associated with mortality risk among colorectal cancer (CRC) patients from two prospective epidemiological cohorts. Results indicated that individuals with clinical depression had higher overall mortality risk after adjustment for relevant potential covariates. Clinical anxiety was also associated with increased risk, although the estimate did not reach statistical significance. This slight difference between anxiety and depression might reflect possible misclassification because physician-diagnosed anxiety was not reported, or the smaller sample size in these analyses. When considering a continuous symptom measure, each 1-SD increase in anxiety or depression symptoms was significantly and comparably associated with elevated mortality risk. Overall, given the similarity of findings across these two forms of distress, results may suggest a transdiagnostic phenomenon, whereby common mental disorders or their subclinical symptoms relate similarly to CRC survival. 

The magnitude of these estimates is meaningful, as it suggests clinically depressed CRC patients have a 28% excess mortality risk compared to their non-clinically depressed counterparts. Moreover, findings from models with continuous symptoms indicate elevated symptoms, even subclinical, would translate into a 16% greater mortality risk. These effect sizes are comparable to those obtained in prior studies [10,12] and only slightly smaller than those of established cancer survival risk factors. Remarkably, these associations were observed independently of cancer characteristics, sociodemographics, cardiometabolic diseases, and lifestyle. While finding comparable associations across women and men was consistent with previous research [16,17], similar associations by cancer stage was somewhat surprising, given prior work suggesting that psychological determinants become less influential as carcinogenesis progresses, because biological factors play a greater role [16]. However, only 0–7% of included participants had metastases, which may limit our ability to observe effect modification. Our findings were also similar when considering overall or CRC-specific mortality, suggesting distress’ association with survival may not solely be due to other conditions, like cardiovascular death [11,15], and despite more prevalent cardiometabolic diseases among clinically anxious and depressed participants at baseline. Another larger study (*n* = 79,079) in which patients with versus without a mood disorder, as extracted from Medicare claims data, also had comparable 14-year risk estimates of overall and colon cancer-specific mortality; however, mood disorder was determined prior to rather than after the cancer diagnosis and anxiety was not evaluated separately [44]. 

Various mechanisms may explain the observed distress-mortality association. A less healthy lifestyle, as defined by engaging in fewer healthy behaviors and having an unhealthy BMI, is related to having more distress symptoms [45] and with reduced subsequent overall and cancer-specific survival [46] among CRC patients. In our study, accounting for lifestyle at baseline and 4 years later did not alter the relationship of anxiety or depression with mortality, suggesting behavioral factors are not a primary pathway. Future research should consider medical compliance, as CRC patients experiencing psychological distress are at greater risk of not initiating/maintaining oncological treatments [44]. Alternatively, biological evidence shows high levels of distress can impact the sympathetic nervous system and hypothalamic-pituitary-adrenal axis function, which in turn can alter the immune response and inflammation processes involved in carcinogenesis progression [15,47] and fatal outcomes like cardiometabolic diseases [48]. Early research further hints to the role of other neuroendocrine hormones and neuropeptides (e.g., oxytocine, dopamine) [47], and transcription factors causing chemotherapeutic resistance [49]. Because of their preliminary nature, these findings on potential psychophysiological pathways relating psychological distress to cancer-related outcomes first warrant replication. If found to be robust in future studies, subsequent research should then examine specific time periods in the etiology of cancer during which they might be more potent [47]. 

This study has some limitations. Anxiety and depression levels were reported by participants rather than clinicians. However, health professionals likely report health information more accurately than participants from the general population [50] and using self-reported scales provides subclinical levels of symptomatology not available from binary clinician diagnoses. Moreover, psychological symptoms were assessed only once, which may overlook changes in symptomatology, particularly those that may occur closer to cancer diagnosis as noted in previous studies [51,52]. Yet, we considered longer-term experience of distress in the first few years following cancer diagnosis, as it may be more representative of patients’ mental health over time than distress acutely felt around the time of diagnosis. Furthermore, prior research on the distress-cancer mortality relationship specifically showed similar or stronger estimates with time-updated exposures across the first 15 months following cancer diagnosis [53], and previous work in the Nurses’ Health Study II, a sister cohort, indicated fairly stable anxiety or depression levels over 1 to 9 years [54]. Therefore, it seems unlikely that having more than one assessment of anxiety or depression symptoms, with some being closer to diagnosis, would have led to drastically different results. 

Additionally, because of the relatively small sample sizes, we did not have a sufficient number of participants to distinguish each cancer stage and had to collapse them into advanced (stages III-IV) and non-advanced (0-II) categories. Neither cohort queried information on the context in which the diagnosis was determined (e.g., via routine screening versus symptom-based); yet, because the method of detection is related to cancer stage and tumor location [55,56] which were already accounted for in our analyses, further controlling for the identification method is unlikely to alter substantially the current results. Similarly, oncological treatments information was not available, but because treatments extend survival, not controlling for these covariates potentially leads to underestimating the associations of interest [13,16]. Lastly, while the homogeneity of our predominantly white professional adults’ samples limits generalizability to other populations, it enhances internal validity. 

The current study has several strengths. Since participants were from two long-running and well-characterized prospective cohorts, we were able to leverage existing data to classify exposures comprehensively, based on multiple relevant indicators (symptom scales, physician diagnosis, medication); we also considered numerous potential confounders and mechanistic pathways relevant to the distress-cancer mortality association. Inverse probability weighting was implemented to reduce concerns about selection/immortal bias. Given the prolonged follow-up periods, we were able to capture longer-term processes like carcinogenesis progression and to lag our analyses to address concerns about reverse causation. Altogether, findings suggest higher anxiety and depression are related to increased mortality risk in CRC patients over a 28-year period, independent of sociodemographics, cancer characteristics, health status, and lifestyle. Identifying upstream modifiable factors may provide novel opportunities to produce improved outcomes, which appears particularly relevant for CRC patients who may still experience substantial distress months to years after diagnosis [51,52,57]. If replicated, the current results imply that assessing, monitoring, and treating anxiety and depression symptoms in CRC patients may not only enhance mental health and quality of life, but also has the potential to improve survival rates. 

## Figures and Tables

**Table 1:** Distribution of age-standardized characteristics of NHS women within four years after CRC diagnosis by level of anxiety or depression.

	Clinical Anxiety Levels		Clinical Depression Levels	
	No (*n* = 238)	Yes (*n* = 97)	No (*n* = 686)	Yes (*n* = 207)
Age, mean (SD) ^§^	74.9 (7.0)	73.8 (6.1)	71.7 (7.4) *	73.1 (7.9) *
Married/partnered, %	60.4	63.4	64.4 *	57.1 *
Registered nurses degree education level, %	70.6	80.5	73.7	75.0
Census tract income, mean (SD)	62,023.1 (21,295.4)	59,505.9 (21,061.5)	63,415.4 (22,800.3)	61,711.3 (22,874.2)
Prevalent cardiometabolic disease, % ^§§^	25.9 *	46.1 *	24.0 *	41.1 *
Lifestyle score, mean (SD) ^§§§^	2.4 (1.1)	2.3 (1.1)	2.3 (1.1) *	2.0 (1.0) *
Age at diagnosis, mean (SD)	72.5 (7.0)	72.7 (6.5)	70.0 (7.5)	70.4 (7.9)
Proximal tumor location	40.6	39.8	48.4	41.8
Advanced cancer (stages III-IV), % ^§§§§^	30.7	28.2	32.3	35.1
Time in years between diagnosis and analytic baseline, median (interquartile range)	2.2 (1.1–3.2)	2.0 (0.9–2.8)	1.9 (1.0–3.1)	1.8 (0.9–2.6)

Notes. ^§^ Value is not age-adjusted; ^§§^ Includes diabetes, myocardial infarction, angina, stroke; ^§§§^ Index of physical activity, diet, body mass index, alcohol and tobacco consumption with higher scores indicating healthier lifestyle; ^§§§§^ Among participants with available data (92–95% of the samples). * Statistically different between clinically relevant versus non-clinically relevant levels of psychological symptoms, *p* ≤ 0.05. CRC=colorectal cancer; NHS = Nurses’ Health Study. Values are means (SD) or percentages and are standardized to the age distribution of the study population. Values of polytomous variables may not sum to 100% due to rounding.

**Table 2:** Distribution of age-standardized characteristics of HPFS men within four years after CRC diagnosis by level of anxiety or depression.

	Clinical Anxiety Levels		Clinical Depression Levels	
	No (*n* = 181)	Yes (*n* = 51)	No (*n* = 225)	Yes (*n* = 47)
Age, mean (SD) ^§^	70.9 (9.9)	72.2 (9.8)	74.5 (8.7)	77.0 (8.8)
Married/partnered, %	90.5	94.4	87.5	79.2
Profession				
Dentist, %	56.8	64.5	63.1	54.8
Osteopath, %	5.5	1.3	4.9	6.5
Pharmacist, %	10.2	11.2	7.0	11.9
Veterinarian, %	15.4	11.0	17.3	22.1
Other (optometrist or podiatrist), %	12.0	12.1	7.6	4.7
Prevalent cardiometabolic disease, % ^§§^	27.3	34.9	30.2	40.3
Lifestyle score, mean (SD) ^§§§^	2.5 (1.1)	2.5 (1.1)	2.5 (1.1) *	2.1 (1.2) *
Age at diagnosis, mean (SD)	69.1 (9.5)	68.6 (10.1)	72.3 (8.8)	72.5 (8.7)
Proximal tumor location	63.9	56.2	66.3	59.7
Advanced cancer (stages III-IV), % ^§§§§^	30.3	32.9	31.5	40.7
Time in years between diagnosis and analytic baseline, median (interquartile range)	1.4 (0.8–2.6)	1.3 (0.8–2.4)	2.3 (1.1–3.0)	2.3 (1.3–3.0)

Notes. ^§^ Value is not age adjusted; ^§§^ Includes diabetes, myocardial infarction, angina, stroke; ^§§§^ Index of physical activity, diet, body mass index, alcohol and tobacco consumption with higher scores indicating healthier lifestyle; ^§§§§^ Among participants with available data (81–82% of the samples). * Statistically different between clinically relevant versus non-clinically relevant levels of psychological symptoms, *p* < 0.05. CRC = colorectal cancer; HPFS = Health Professional Follow-up Study. Values are means (SD) or percentages and are standardized to the age distribution of the study population. Values of polytomous variables may not sum to 100% due to rounding.

**Table 3:** Meta-analysis of the association of post-diagnosis anxiety and depression symptoms with overall mortality risk over up to 28 years of follow-up in the NHS and HPFS cohorts.

	Anxiety		*p*-Value for Heterogeneity	Depression^§^		*p*-Value for Heterogeneity
	HR	95% CI		HR	95% CI	
	Sample Size (Number of Deaths/Person-Years)			Sample Size (Number of Deaths/Person-Years)		
Continuous distress level (standardized; per 1-SD)	*n* = 567 (270/4667)			*n* = 1009 (458/8718)		
Model 1	1.17 ^**^	1.06–1.30	0.63	1.20 ^****^	1.11–1.31	0.003
Model 2	1.17 ^**^	1.05–1.30	0.90	1.17 ^****^	1.08–1.27	0.002
Model 3	1.16 ^**^	1.05–1.29	0.92	1.16 ^***^	1.07–1.26	0.003
						
Dichotomized distress level (clinical versus non-clinical)	Clinical level: *n* = 148 (82/1261) Non-clinical level: *n* = 419 (188/3406)			Clinical level: *n* = 254 (128/1814) Non-clinical level: *n* = 911 (416/8223)		
Model 1	1.22	0.96–1.55	0.29	1.38 ^***^	1.14–1.66	0.25
Model 2	1.17	0.91–1.49	0.45	1.31 ^**^	1.08–1.59	0.12
Model 3	1.17	0.92–1.50	0.47	1.28 ^**^	1.06–1.56	0.14

Notes. ** *p* ≤ 0.01, *** *p* ≤ 0.001, **** *p* ≤ 0.0001. CI = Confidence Intervals; HR = Hazard Ratio; HPFS = Health Professional Follow-up Study; NHS = Nurses’ Health Study. ^§^ See Text S4 for information about the derivation of the analytic sample sizes used in these analyses. **Model 1:** Adjusted for age at diagnosis (continuous), year at diagnosis (continuous), cancer stage (advanced versus non-advanced (III-IV versus 0-II)), missing indicator for cancer stage, tumor location (“proximal colon” versus “distal colon/rectal”), and time between diagnosis and analytic baseline (i.e., anxiety/depression assessment; continuous). **Model 2**: Model 1 + age at analytic baseline (i.e., anxiety/depression assessment; continuous), census track income (NHS; continuous), education (NHS; registered nurses versus university degree), occupation (HPFS; dentist versus osteopath versus pharmacist versus veterinarian versus other [optometrist/podiatrists]), and prevalent cardiometabolic disease (i.e., diabetes, myocardial infarction, angina, stroke; yes/no). **Model 3:** Model 2 + lifestyle score (i.e., index of physical activity, diet, body mass index, alcohol and tobacco consumption; continuous) at analytic baseline (i.e., anxiety/depression assessment; continuous).

**Table 4:** Association of post-diagnosis anxiety and depression symptoms with mortality risk over up to 28 years of follow-up by cohort.

	Anxiety		Depression ^§^	
	HR	95% CI	HR	95% CI
	Sample Size (Number of Deaths/Person-Years)		Sample Size (Number of Deaths/Person-Years)	
	**Women (NHS)**			
Continuous distress levels (standardized; per 1-SD)	*n* = 335 (122/2346)		*n* = 887 (412/8090)	
Model 1	1.21 ^**^	1.04–1.40	1.15 ^***^	1.06–1.26
Model 2	1.17 ^*^	1.01–1.37	1.12 ^**^	1.03–1.22
Model 3	1.17 ^*^	1.00–1.36	1.12 ^**^	1.02–1.22
				
Dichotomized distress levels (clinical versus non-clinical)	Clinical level: *n* = 97 (45/743) Non-clinical level: *n* = 238 (77/1603)		Clinical level: *n* = 207 (103/1573) Non-clinical level: *n* = 686 (313/6551)	
Model 1	1.37 ^†^	0.99–1.90	1.30 ^*^	1.05–1.61
Model 2	1.28	0.91–1.79	1.22 ^†^	0.98–1.51
Model 3	1.28	0.91–1.80	1.19	0.96–1.48
	**Men (HPFS)**			
Continuous distress levels (standardized; per 1-SD)	*n* = 232 (148/2321)		*n* = 122 (46/628)	
Model 1	1.15 ^†^	0.99–1.32	1.70 ^****^	1.34–2.17
Model 2	1.16 ^*^	1.00–1.34	1.70 ^****^	1.33–2.19
Model 3	1.16 ^*^	1.00–1.34	1.68 ^****^	1.30–2.18
Dichotomized distress levels (clinical versus non-clinical)	Clinical levels: *n* = 51 (37/518) Non-clinical level: *n* = 181 (111/1803)		Clinical: *n* = 47 (25/241) Non-clinical level: *n* = 225 (103/1672)	
Model 1	1.06	0.74–1.51	1.72 ^**^	1.13–2.62
Model 2	1.06	0.74–1.51	1.77 ^**^	1.16–2.71
Model 3	1.07	0.74–1.53	1.72 ^**^	1.11–2.64

Notes. ^†^ *p* ≤ 0.10, * *p* ≤ 0.05, ** *p* ≤ 0.01, *** *p* ≤ 0.001, **** *p* ≤ 0.0001. CI = Confidence Intervals; HR = Hazard Ratio; HPFS = Health Professional Follow-up Study; NHS = Nurses’ Health Study. ^§^ See Text S4 for information about the derivation of the analytic sample sizes used in these analyses, **Model 1:** Adjusted for age at diagnosis (continuous), year at diagnosis (continuous), cancer stage (advanced versus non-advanced [III-IV versus 0-II]), missing indicator for cancer stage, tumor location (“proximal colon” versus “distal colon/rectal”), and time between diagnosis and analytic baseline (i.e., anxiety/depression assessment; continuous). **Model 2**: Model 1 + age at analytic baseline (i.e., anxiety/depression assessment; continuous), census track income (NHS; continuous), education (NHS; registered nurses versus university degree), occupation (HPFS; dentist versus osteopath versus pharmacist versus veterinarian versus other [optometrist/podiatrists]), and prevalent cardiometabolic disease (i.e., diabetes, myocardial infarction, angina, stroke; yes/no). **Model 3:** Model 2 + lifestyle score (i.e., index of physical activity, diet, body mass index, alcohol and tobacco consumption; continuous) at analytic baseline (i.e., anxiety/depression assessment; continuous).

## Supplementary Materials

The following are available online at https://www.mdpi.com/2077-0383/9/10/3174/s1, Figure S1: Flowchart of the analytic samples in both cohorts; Figure S2: Timeline of anxiety and depression assessments in both cohorts, Table S1: Comparison between participants who did versus did not complete an anxiety or depression assessment within 4 years after receiving a diagnosis of colorectal cancer on post-diagnosis demographic and medical characteristics; Table S2: Fully-adjusted models evaluating the association of post-diagnosis anxiety and depression symptoms with mortality risk over up to 28 years of follow-up while considering lifestyle assessed 4 years after baseline as a potential pathway, Text S1: Additional information about the anxiety and depression self-reported symptom measures; Text S2: Consideration of medication use in analyses using a continuous exposure; Text S3: Creation of the lifestyle score; Text S4: Derivation of the analytic sample sizes.

## Data Accessibility

The data that support the findings of our study are available from Brigham and Women’s Hospital and Harvard T.H. Chan School of Public Health. Restrictions apply to the availability of these data, which were used under license for our study. Data are available (https://sites.google.com/channing.harvard.edu/cohortdocs/) with the permission of Brigham and Women’s Hospital and Harvard T.H. Chan School of Public Health.

## Abbreviations

BMI	Body Mass Index
CI	Confident Interval
CRC	Colorectal Cancer
HPFS	Health Professional Follow-Up Study
HR	Hazard Ratio
M	Mean
NHS	Nurses’ Health Study
SD	Standard Deviation
