# Supplementary material for: Anxiety, Depression, and Colorectal Cancer Survival: Results from Two Prospective Cohorts
Source: J Clin Med. 2020 Sep 30;9(10):3174. doi: 10.3390/jcm9103174 (PMC7599619; doi:10.3390/jcm9103174)
Supplement: Supplementary file 1 [file jcm-09-03174-s001.pdf]

Supplementary Materials

Anxiety, Depression, and Colorectal Cancer Survival: Results from Two Prospective Cohorts

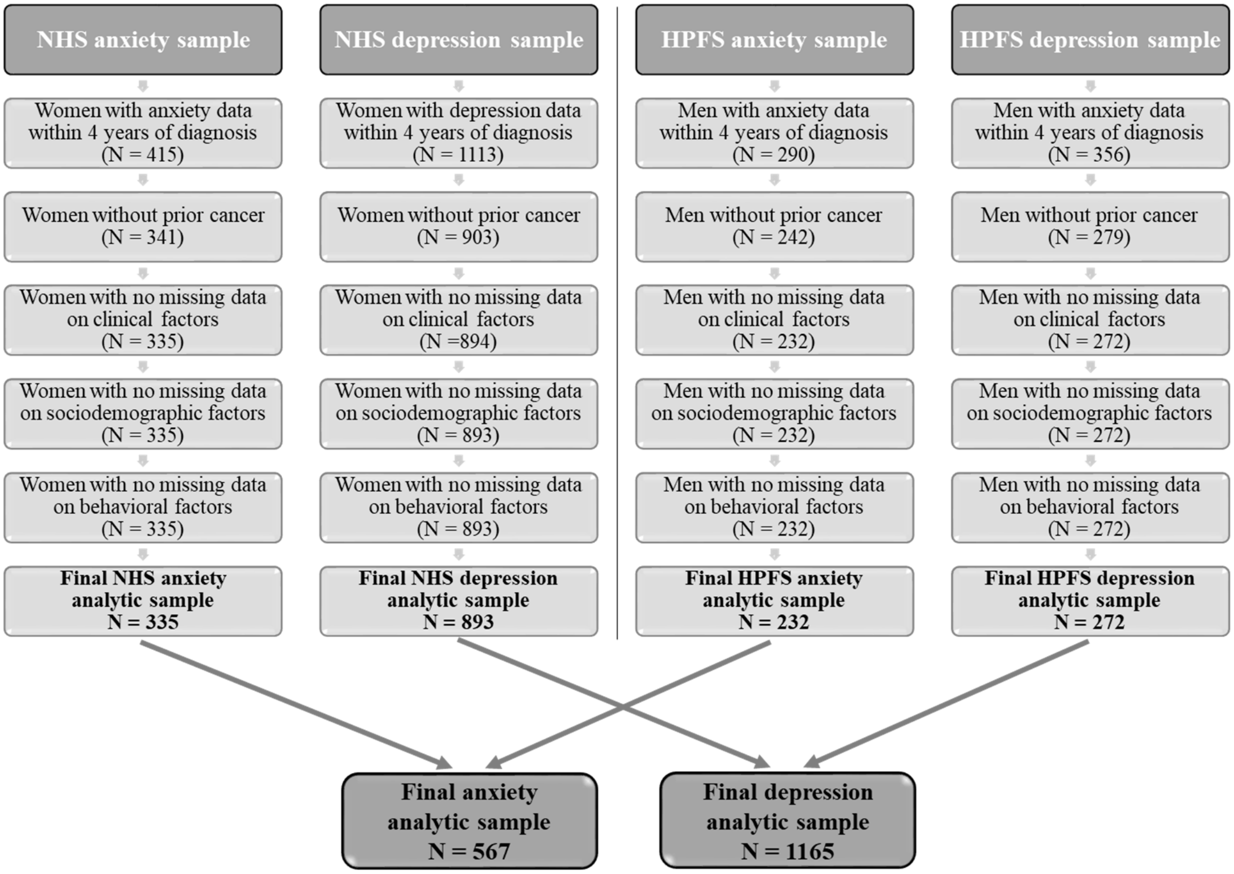

Figure S1. Flowchart of the analytic samples in both cohorts.

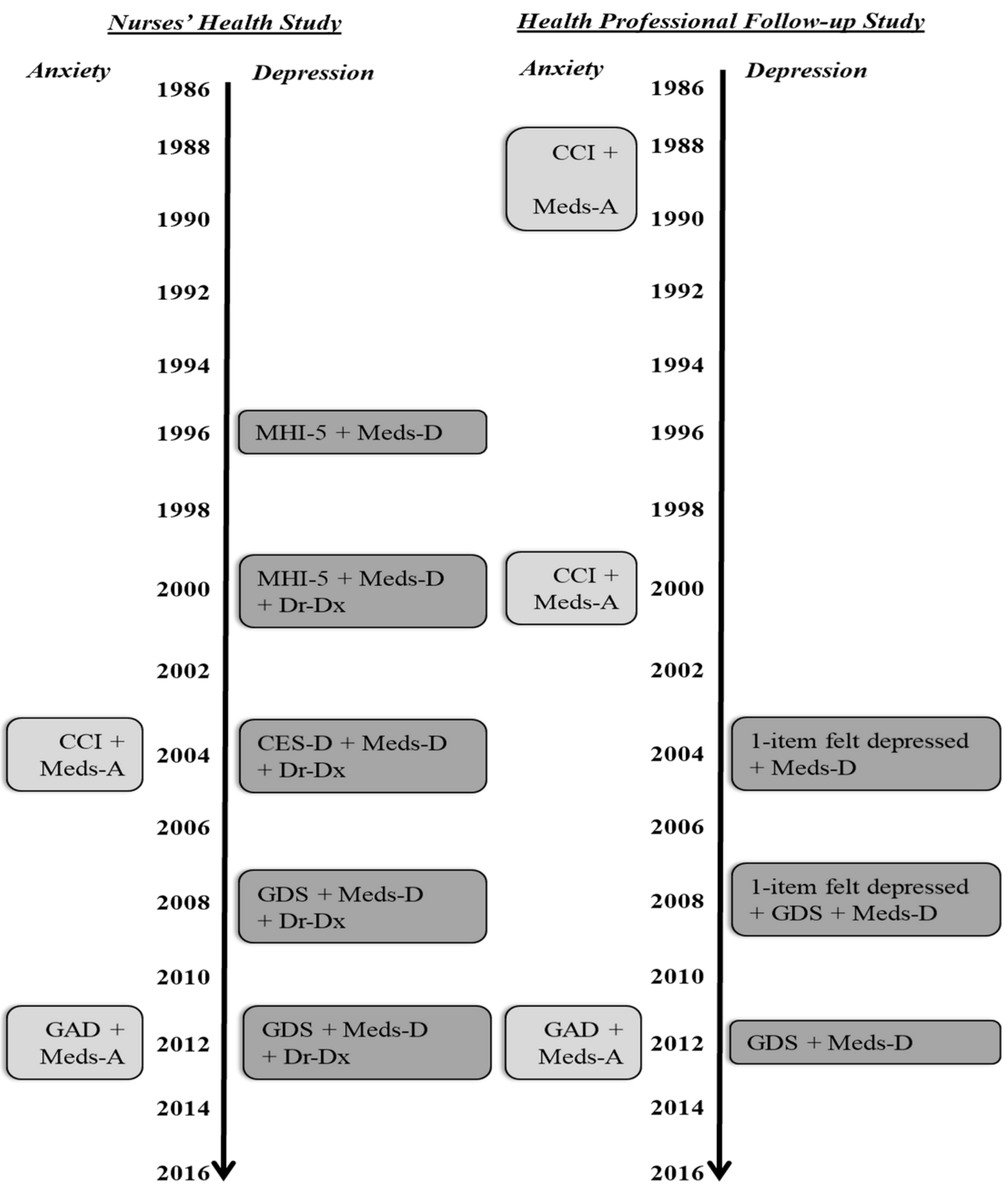

Figure S2. Timeline of anxiety and depression assessments in both cohorts. *Notes.* Individuals who completed an anxiety or depression assessment within 4 years after receiving a diagnosis of colorectal cancer were included in the study. These assessments define the analytic baselines to be pooled within each cohort. No participants from the anxiety analytic samples overlapped with those of the depression analytic samples, except for the 2004 assessment in NHS (n = 205) and the 2012 assessment in both cohorts (NHS: n = 125; HPFS: n = 45), which represents 22% (375/1,732) of the total study sample. Because no anxiolytic assessment was conducted in 1988 among HPFS men, anxiolytic use from 1990 was used in the current study. Measures of anxiety, on the left side of each cohort, with CCI = Crown-Crisp Index, GAD=Generalized Anxiety Disorder, and Meds-A = anxiolytics. Measures of depression, on the right side of each cohort, with CES-D = Center for Epidemiologic Studies–Depression, Dr-Dx = Doctor-diagnosed depression, GDS = Geriatric Depression Scale, Meds-D = antidepressants; MHI-5 = Mental Health Index-5 items from the SF-36 survey, 1-item screener about feeling depressed.

Table S1. Comparison between participants who did/did not complete an anxiety or depression assessment within 4 years after receiving a colorectal cancer diagnosis on post-diagnosis demographic and medical characteristics.

	**Nurses' Health Study (NHS): Anxiety**		
	**Completed assessment (*n* = 415)**	**Did not complete assessment (*n* = 269)**	***p*-value**
Age (mean; SD)	74.9 (6.7)	76.5 (7.0)	0.003
Education (% University degree)	28.7	17.1	0.001
Marital status (% in a relationship)	58.6	61.9	0.41
Prevalent cardiometabolic disease (% yes)	32.5	64.4	≤0.0001
Cancer stage (% advanced)	28.0	55.4	≤0.0001
Tumor location (% proximal colon)	60.2	52.7	0.06
Baseline lifestyle score (mean; SD)	2.4 (1.1)	2.1 (1.1)	0.001
	**Nurses' Health Study (NHS): Depression**		
	**Completed assessment (*n* = 1,113)**	**Did not complete assessment (*n* = 702)**	***p*-value**
Age (mean; SD)	72.3 (7.6)	73.2 (8.1)	0.01
Education (% University degree)	26.6	16.0	≤0.0001
Marital status (% in a relationship)	62.4	70.1	0.001
Prevalent cardiometabolic disease (% yes)	28.8	25.5	0.12
Cancer stage (% advanced)	29.7	53.9	≤0.0001
Tumor location (% proximal colon)	55.0	49.3	0.02
Baseline lifestyle score (mean; SD)	2.3 (1.1)	2.1 (1.0)	0.003
	**Health Professional Follow-Up Study (HPFS): Anxiety**		
	**Completed assessment (*n* = 290)**	**Did not complete assessment (*n* = 186)**	***p*-value**
Age (mean; SD)	72.8 (9.9)	75.5 (10.5)	0.01
Education			0.25
Dentist, %	59.3	58.1	
Osteopath, %	3.8	6.5	
Pharmacist, %	10.0	5.4	
Veterinarian, %	16.2	16.7	
Other (optometrist or podiatrist), %	10.7	13.4	
Marital status (% in a relationship)	89.3	87.6	0.57
Prevalent cardiometabolic disease (% yes)	30.3	54.9	≤0.0001
Cancer stage (% advanced)	24.5	40.9	0.0002
Tumor location (% proximal colon)	39.7	45.0	0.31
Baseline lifestyle score (mean; SD)	2.4 (1.1)	2.4 (1.2)	0.65
	**Health Professional Follow-Up Study (HPFS): Depression**		
	**Completed assessment (*n* = 356)**	**Did not complete assessment (*n* = 224)**	***p*-value**
Age (mean; SD)	76.1 (8.7)	79.6 (8.9)	≤0.0001
Education (% University degree)			0.86
Dentist, %	61.2	61.6	
Osteopath, %	3.9	5.8	
Pharmacist, %	8.2	7.1	
Veterinarian, %	18.0	17.4	
Other (optometrist or podiatrist), %	8.7	8.0	
Marital status (% in a relationship)	84.8	87.6	0.38
Prevalent cardiometabolic disease (% yes)	33.2	71.8	≤0.0001
Cancer stage (% advanced)	27.0	32.1	0.18
Tumor location (% proximal colon)	38.2	50.7	0.01
Baseline lifestyle score (mean; SD)	2.4 (1.1)	2.5 (1.3)	0.61

Notes. Age, marital status and lifestyle were measured at baseline, when psychological symptoms were queried (NHS: 2004, 2012 for anxiety; 1996, 2000, 2004, 2008, 2012 for depression/HPFS: 1988, 2000, 2012 for anxiety; 2004, 2008, 2012 for depression), while the other characteristics were time invariant. Significant differences were verified with t-tests for age and lifestyle, and with chi-square tests for education, marital status, cardiometabolic disease (i.e., diabetes or cardiovascular disease), cancer stage, and tumor location. To compare participants who did vs. did not complete the anxiety or depression assessment, the vast majority of covariates had no missing data. However, in some samples, age (HPFS: depression = 0.2%), marital status (NHS: anxiety = 4.8%/ HPFS: depression = 5.3%), prevalent cardiometabolic disease (NHS: anxiety = 22.1% / HPFS: anxiety = 17.7%; depression = 18.5%), baseline lifestyle score (NHS: anxiety = 7.2%), cancer stage (NHS: anxiety = 11.3%, depression = 13.0%/HPFS: anxiety = 25.8%; depression = 28.6%), and tumor location (NHS: anxiety = 1.9%, depression = 2.2%/HPFS: anxiety = 12.0%; depression = 12.4%) data was missing. In these cases, the comparisons are based on participants with data only.

Table S2. Fully-adjusted models evaluating the association of post-diagnosis anxiety and depression symptoms with mortality risk over up to 28 years of follow-up while considering lifestyle assessed 4 years after baseline as a potential pathway.

	Anxiety		Depression§	
	HR	95% CI	HR	95% CI
	Sample size (number of deaths/person-years)		*Sample size (number of deaths/person-years)*	
	**Meta-analysis of both cohorts**			
Continuous symptoms levels (standardized; per 1-SD)	*n = 394 (224/4069)*		(no available data in HPFS)	
Fully-adjusted including baseline lifestyle	**1.20****	**1.06–1.35**		
Further adjustment for lifestyle 4 years later	**1.20****	**1.07–1.35**		
Dichotomized symptoms levels (clinical vs. non-clinical)	*Clinical level: n = 129 (77/1198)*<br>*Non-clinical level: n = 265 (147/2871)*		*Clinical level: n = 123 (66/1243)*<br>*Non-clinical level: n = 615 (324/6628)*	
Fully-adjusted including baseline lifestyle	1.14	0.88–1.49	1.09	0.84–1.41
Further adjustment for lifestyle 4 years later	1.17	0.90–1.53	1.09	0.84–1.41
	**Women (NHS)**			
Continuous symptoms levels (standardized; per 1-SD)	*n = 209 (91/1912)*		*n = 586 (306/6578)*	
Fully-adjusted including baseline lifestyle	**1.25***	**1.04–1.50**	1.08	0.97–1.20
Further adjustment for lifestyle 4 years later	**1.27****	**1.06–1.52**	1.08	0.97–1.20
Dichotomized symptoms levels (clinical vs. non-clinical)	*Clinical level: n = 83 (41/697)*<br>*Non-clinical level: n = 126 (50/1215)*		*Clinical level: n = 112 (62/1148)*<br>*Non-clinical level: n = 478 (248/5451)*	
Fully-adjusted including baseline lifestyle	1.26	0.86–1.85	1.10	0.84–1.43
Further adjustment for lifestyle 4 years later	1.34	0.91–1.97	1.10	0.84–1.44

Table S2. *Cont.*

	Men (HPFS)			
Continuous symptoms levels<br>(standardized; per 1-SD)	*n = 185 (133/2156)*			
Fully-adjusted including baseline lifestyle	1.16†	0.99–1.35	(no available data)	
Further adjustment for lifestyle 4 years later	1.16†	0.99–1.35		
Dichotomized symptoms levels<br>(clinical vs. non-clinical)	*Clinical level: n = 46 (36/501)*<br>*Non-clinical level: n = 139 (97/1655)*		*Clinical level: n = 11 (4/95)*<br>*Non-clinical level: n = 137 (76/1177)*	
Fully-adjusted including baseline lifestyle	1.04	0.72–1.51	1.01	0.38–2.67
Further adjustment for lifestyle 4 years later	1.04	0.72–1.51	1.01	0.38–2.69

Notes. $^{\dagger}p \leq 0.10$, $^{*}p \leq 0.05$, $^{**}p \leq 0.01$, $^{***}p \leq 0.001$, $^{****}p \leq 0.0001$. CI = Confidence Intervals; HR = Hazard Ratio; HPFS = Health Professional Follow-up Study; NHS = Nurses' Health Study. [§] See Text S4 for information about the derivation of the analytic sample sizes used in these analyses. **Model with lifestyle** at baseline only: Adjusted for age at diagnosis (continuous), year at diagnosis (continuous), cancer stage (advanced vs. non-advanced (III-IV vs. 0-II)), missing indicator for cancer stage, tumor location ("proximal colon" vs. "distal colon/rectal"), time between diagnosis and analytic baseline (i.e., anxiety/depression assessment; continuous), age at analytic baseline (i.e., anxiety/depression assessment; continuous), census track income (NHS; continuous), education (NHS; registered nurses vs. university degree), occupation (HPFS; dentist vs. osteopath vs. pharmacist vs. veterinarian vs. other (optometrist/podiatrists)), prevalent cardiometabolic disease (i.e., diabetes, myocardial infarction, angina, stroke; yes/no), lifestyle score (i.e., index of physical activity, diet, body mass index, alcohol and tobacco consumption; continuous) at analytic baseline (i.e., anxiety/depression assessment; continuous). Model with lifestyle at baseline and 4 years later: Model described above + lifestyle score (i.e., index of physical activity, diet, body mass index, alcohol and tobacco consumption; continuous) 4 years after exposure assessment.

Text S1. Additional information about the anxiety and depression self-reported symptom measures.

Anxiety symptoms were assessed in 2004 in NHS, as well as in 1988 and 2000 in HPFS using the validated self-report 8-item Crown-Crisp Index (CCI) [1] as well as in 2012 in both cohorts using the validated self-report 7-item Generalized Anxiety Disorder (GAD-7) [2]. Although the CCI focuses mostly on symptoms relevant to fear and phobia disorders like panic disorder and agoraphobia (e.g., feeling panicky in crowds), it also incorporates worry-related items (e.g., worrying unduly when relatives are late coming home) [1,3]. The eight items are scored as 0 "never", 1 "sometimes", or 2 "always", with a derived sum ranging from 0 (no anxiety) to 16 (high anxiety). The CCI has been validated in psychiatric outpatient settings and found to discriminate patients with anxiety from healthy controls [1,4]. Internal consistency reliability in the cohorts is modest (NHS: α_{2004} = 0.57; HPFS: α_{1988} = 0.47 α_{2000} = 0.50). Anxiety symptoms obtained with the CCI were dichotomized into high (≥4) vs. lower (<4) symptom levels following prior work on cut points that suggest clinical anxiety levels [5]. The GAD-7 items assess the frequency to which individuals have been bothered by various anxiety symptoms over the past 2 weeks and are scored as 0 "not at all", 1 "several days", 2 "over half the days", or 3 "nearly every day." The derived sum ranges from 0 (no anxiety) to 21 (high anxiety) and was dichotomized into high (≥10) vs. lower (<10) symptom levels.[2] In both cohorts, the internal consistency is very good (NHS: α_{2012} = 0.87; HPFS: α_{2012} = 0.86).

Despite CCI's somewhat lower internal consistency, its standardized scores are moderately correlated with those obtained from other validated scales administered later in both cohorts. These other scales include the GAD-7 (described above) [2] and a modified 10-item version of the State-Trait Anxiety Inventory (STAI), with scores ranging from 0–30 [6]. Using the closest time assessments in each cohort among the full cohort populations (n's range from 20,618 to 72,813); in NHS: CCI_{2004} with $STAI_{2004}$, r = 0.41, p < 0.0001 and CCI_{2004} with GAD-7_{2012}, r = 0.31, p < 0.0001; in HPFS: CCI_{2000} with GAD-7_{2012}, r = 0.25, p < 0.0001. Moreover, using the two time assessments in HPFS, the intra-class correlation coefficient indicates that anxious symptoms are fairly stable in this cohort (intra-class correlation (ICC) = 0.59).

Within NHS, three different instruments have been used to assess depressive symptoms. In 1996 and 2000, the 5-item Mental Health Index (MHI-5) from the Medical Outcomes Study Short-Form 36 Health Status Survey [7] was queried regarding symptoms occurring in the past month. Items response options ranged from 1 "all the time" to 6 "none of the time," and item scores were summed and converted to a 100 point scale for a total score with the highest score indicating the lowest symptom levels. Following previous work identifying cut points suggesting clinical levels of depression [8], the total score was dichotomized with ≤60 indicating higher and >60 lower depression symptoms. The MHI-5 has good predictive value for detecting mood disorders/major depression [9,10]. In the NHS cohort, the scale has a high internal consistency reliability (α_{1996}=0.81; α_{2000}=0.80) and scores are reasonably stable over the two assessments (ICC = 0.57). In 2004, a modified 10-item version of the Center for Epidemiologic Studies-Depression Scale (CES-D) [11] measured depression symptoms that occurred in the past month. Respondents rated the items on a 4-point scale, ranging from 0 "Rarely or none of the time," to 3 "All of the time," and items were summed for a total score from 0 to 30. Internal consistency reliability is good in NHS (α = 0.78). As determined in the CES-D initial validation study identifying a score suggestive of clinical depression [11], the score was dichotomized with ≥10 indicating higher and <10 lower depression symptoms. In 2008 and 2012, the 15-item Geriatric Depression Scale-Short Form (GDS-SF) [12] was administered, to focus on the unique aspects of geriatric depression. Binary items are rated 0 "No; symptom absent" or 1"Yes; symptom present" in the last week. Following previous work identifying a clinical depression cutpoint among cancer patients, the total score was dichotomized with ≥4 indicating higher and <4 lower depression symptoms [13]. Within HPFS, the GDS-SF was also used in 2008 and 2012 to measure depressive symptoms, using the same cutpoint. In both cohorts, the GDS-SF has high internal consistency reliability (NHS: α_{2008}=0.80, α_{2012}=0.81; HPFS: α_{2008} = 0.74, α_{2012} = 0.75), with fairly stable scores across the two assessments (NHS: ICC = 0.65; HPFS:

ICC = 0.56). In HPFS, depression was also measured using a screening item [14] in 2004 and 2008, on which men reported whether, within the last 2 years, they had ≥2 weeks when nearly every day they felt sad, blue, or depressed for most of the day (yes/no).

Besides all having high levels of internal consistency, all depression measures queried in the NHS cohort are substantially correlated. For instance, using the standardized scores of the closest assessments among the full NHS cohort (n's range from 63,436 to 69,260): MHI-5_{2000} with CES-D_{2004}, $r = 0.50$ ($p < 0.0001$); CES-D_{2004} with GDS-SF_{2008}, $r = 0.50$ ($p < 0.0001$); and MHI-5_{2000} with GDS-SF_{2008}, r=0.38 (p<.0001). In a more thorough investigation of their comparability among NHS women, scores from these three scales were found to be highly consistent based on the equipercentile equating method [15]. The scores of these scales are also correlated with self-reported physician-diagnosis of depression in the NHS cohort (e.g., CES-D_{2004} with physician-diagnosed_{2004}, point-biserial correlation $r_{pb} = 0.26$, $p < 0.0001$; GDS_{2008} with physician-diagnosed_{2008}, $r_{pb} = 0.25$, $p < 0.0001$).

While measures of both anxiety and depression are available in our samples, they were mainly taken at different time points which led to different analytic samples based on which measures were completed within four years following respondents' CRC diagnosis. However, it is worth noting that within the larger NHS and HPFS cohorts, anxiety and depression measures are modestly correlated. For example, using standardized scores obtained at the closest time assessments among the full cohorts (n's range from 22,865 to 69,702): in NHS, CCI_{2004} with MHI-5_{2000}, $r = 0.30$ ($p < 0.0001$) and GAD-7_{2012} with GDS_{2012}, $r = 0.51$ ($p < 0.0001$); in HPFS, CCI_{2000} with GDS-SF_{2008}, $r = 0.22$ ($p < 0.0001$) and GAD-7_{2012} with GDS_{2012}, $r = 0.48$ ($p < 0.0001$).

Although psychological symptoms were assessed before CRC diagnosis in the larger NHS/HPFS cohorts, they were not included in the current analyses for two main reasons. First, there is some evidence suggesting that psychological symptoms related to anxiety or depression are generally stable when comparing their levels before versus several years after receiving a diagnosis of a major medical condition, including cancer [16,17]. We are aware that changes in psychological states might occur closer in time, such as in the first year following a cancer diagnosis [17–21]; unfortunately, in the NHS/HPFS cohorts, no measures of anxiety or depression symptoms were obtained closer in time that may capture more subtle or immediate changes in psychological symptoms from pre- to post-diagnosis. Second, only a subset of our participants has information on pre-diagnosis levels of psychological symptoms. For instance, among our current analytic sample of 335 and 232 participants of the NHS and HPFS cohorts, respectively, who had completed a measure of anxiety within 4 years after receiving a colorectal cancer diagnosis, only 116 (35%) and 113 (49%) also had a pre-diagnosis symptom measure of anxiety, respectively. These measures were also obtained from 8 to 12 years before the post-diagnosis symptom measure used as baseline in the current study. Therefore, considering pre-diagnosis level of psychological symptoms would reduce our (already somewhat small) sample sizes without adding valuable information or critical insight on the current findings given the stability of the scores.

Text S2. Consideration of medication use in analyses using a continuous exposure.

Among participants who reported using psychotropic medication, many had a score on the symptom scales that was below the clinical cutpoint (i.e., in NHS, between 53% and 78%; in HPFS, between 38% and 100%), highlighting the potential for misclassification. Because reporting a non-clinical score might in fact be due to the effect of the medication –whereby without medication, these participants would possibly score higher on the self-reported scales–, we evaluated two alternative definitions of the continuous exposures in preliminary analyses. First, to these participants we imputed the mean value of scores that were equal or above the clinical cutpoint in their respective analytic sample. For instance, if the mean value of clinical scores on the Crown Crisp Index was 5.4, we imputed the value of 5.4 to participants who were both using anxiolytics and reporting a non-clinical score on the Crown Crisp Index. Second, to these participants we increased their score on the symptom scale from a specific value that would bring the

lowest score among this subset of participants equal or above the clinical cutpoint. For instance, if the lowest value among these few participants was 1 on the Crown Crisp Index, 3 points were added to the scores of all of these participants so that they all had a score in the clinical range (i.e., equal or above 4 in this case). Because both strategies led to results that were similar to those obtained without imputation, only results based on non-imputed symptom scale scores were reported in the manuscript.

Text S3. Creation of the lifestyle score.

Consistent with a lifestyle index used in previous studies [22–24] as well as with available survivorship cancer guidelines [25,26], the lifestyle score included five behavior-related factors: physical activity, diet, body mass index (BMI), alcohol and tobacco consumption. Individual behaviors were obtained via self-report at each baseline (NHS: 1996, 2000, 2004, 2008, 2012; HPFS: 1988, 2000, 2004, 2008, 2012). Based on prior research [22,24,27], behaviors were first dichotomized according to whether individuals were compliant with recommended guidelines or not (1/0) and then summed to create a lifestyle score, ranging from 0 "least healthy" to 5 "most healthy".

Physical activity was assessed with a self-administered questionnaire that has been developed and validated in the HPFS cohort [28]. It measures the average weekly time spent at various activities, from inactive to vigorous ones, over the past year. The questionnaire has shown high validity when compared against 1-week diaries (e.g., correlation with vigorous activities, r values from 0.54 to 0.58) and resting pulse rate as an objective fitness metric independent of recall (e.g., correlation with vigorous activities, r values from -0.41 to -0.45). Reproducibility over a one-year period was found to be adequate as well (e.g., for vigorous activities, intra-class correlation [ICC] values from 0.32 to 0.79). In the current study, a score of 1 was assigned when women reported ≥150 minutes per week of moderate-to-vigorous activity (e.g., brisk walking, running, bicycling). BMI was derived using participants' self-reported initial height and updated weight at each follow-up assessment after CRC diagnosis. Previous work with the NHS and HPFS cohorts has shown self-reported weight is highly correlated with weight measured by study staff (r = 0.96) [29]. Optimal weight (score of 1) was defined as BMI≤25 kg/m^2. Dietary information was obtained from the 131 item Food Frequency Questionnaire, which has high reproducibility and validity when compared with 1-week diet records and biochemical markers in these cohorts [30,31]. The summary score used here encompasses the following components of the Alternative Healthy Eating Index [32]: higher intake of vegetables, fruit, whole grains, nuts and legumes, long-chain (n-3) fatty acids, polyunsaturated fats; lower intake of sugar-sweetened beverages and fruit juice, red/processed meat, saturated fats, sodium. The score for each component ranged from 0 to 10 (optimal dietary behavior). Within these cohorts, researchers have used the highest 40% of the diet score distribution as a cutoff to indicate healthy diet [33,34], and found it is related to a lower risk of several diseases, including stroke, diabetes and cancer.[35] Accordingly, a healthy diet (score of 1) was defined as a score in the top 40% of the current cohort distribution, updated at each follow-up assessment. Healthy alcohol consumption (score of 1) was defined as drinking ≤1 drink/day [26]. Lastly, participants received a score of 1 if they reported currently being a non-smoker.

Text S4. Derivation of the analytic sample sizes.

S4.1. All models.

The sample sizes for the anxiety and depression analyses vary for the following reason: Continuous exposures were based on standardized anxiety/depression z-scores from self-reported scales, whereas dichotomized exposures were based on having any of: reaching the clinical cutpoint on self-reported scales, using relevant psychotropic medication, or reporting a physician diagnosis (depression only). In this context, the dichotomized depression exposure includes additional information about physician-diagnosed depression and medication (yes/no) that cannot be used for the continuous exposure. For anxiety

exposures, sample sizes are identical because all participants who had information about use of medication necessary for the dichotomized exposure also had completed the self-report symptom scale necessary for creating the continuous exposure.

S4.2. Sensitivity models considering behavioral factors post-baseline as potential pathway.

Because only participants who had data on the lifestyle score four years after analytic baseline (when anxiety and depression were assessed) were included in these secondary analyses, sample sizes are slightly smaller than ones used in the main models: continuous exposures: $N_{anxiety}$ = 394 (209 women/185 men), $N_{depression}$ = 586 (586 women/0 men); dichotomized exposures: $N_{anxiety}$ = 394 (209 women/185 men), $N_{depression}$ = 738 (590 women/148 men)). Lifestyle was not time-updated because i) two out of four analytic samples (i.e., sample for examining anxiety in NHS; sample for examining depression in HPFS) had no further lifetime assessments after the baseline and 4-year post-baseline lifestyle assessment due to the research design; and ii) continuous lifestyle scores were fairly stable in the two other analytic samples (i.e., depression in NHS: intra-class correlation (ICC) = 0.67; anxiety in HPFS: ICC = 0.64). Of note, none of the 122 men included in the models using the continuous depression symptom score had lifestyle data 4 years post-baseline; therefore, these analyses were executed with models using anxiety with the continuous symptom score and also dichotomized according to symptoms and use of medication, and using only depression dichotomized according to symptoms, diagnosis, and medication use.

Supplementary Materials References

1. Crown S, Crisp AH. A short clinical diagnostic self-rating scale for psychoneurotic patients: The Middlesex Hospital Questionnaire (M.H.Q.). *Br J Psychiatry.* 1966;112(490):917-923.
2. Spitzer RL, Kroenke K, Williams JB, Lowe B. A brief measure for assessing generalized anxiety disorder: The GAD-7. *Archives of internal medicine.* 2006;166(10):1092-1097.
3. Trudel-Fitzgerald C, Chen Y, Singh A, Okereke OI, Kubzansky LD. Psychiatric, psychological, and social determinants of health in the Nurses' Health Study cohorts. *American journal of public health.* 2016;106(9):1644-1649.
4. Burgess PM, Mazzocco L, Campbell IM. Discriminant validity of the Crown-Crisp Experiential Index. *Br J Med Psychol.* 1987;60 (Pt 1):61-69.
5. Albert CM, Chae CU, Rexrode KM, Manson JE, Kawachi I. Phobic anxiety and risk of coronary heart disease and sudden cardiac death among women. *Circulation.* 2005;111(4):480-487.
6. Spielberger CD, Gorush RL, Lushene RE. *Manual for the State-Trait Anxiety Inventory.* Palo Alto, CA: Consulting Psychologists Press; 1974.
7. Ware JE, Jr., Sherbourne CD. The MOS 36-item short-form health survey (SF-36). I. Conceptual framework and item selection. *Med Care.* 1992;30(6):473-483.
8. Rumpf HJ, Meyer C, Hapke U, John U. Screening for mental health: validity of the MHI-5 using DSM-IV Axis I psychiatric disorders as gold standard. *Psychiatry Res.* 2001;105(3):243-253.

9. Berwick DM, Murphy JM, Goldman PA, Jr. WJE, Barsky AJ, Weinstein MC. Performance of a five-item mental health screening test. *Med Care.* 1991;29(2):169-176.
10. Cuijpers P, van Straten A, van Schaik A, Andersson G. Psychological treatment of depression in primary care: a meta-analysis. *Br J Gen Pract.* 2009;59(559):e51-60.
11. Andresen EM, Malmgren JA, Carter WB, Patrick DL. Screening for depression in well older adults: Evaluation of a short form of the CES-D (Center for Epidemiologic Studies Depression Scale). *American journal of preventive medicine.* 1994;10(2):77-84.
12. Yesavage JA, Brink TL, Rose TL, et al. Development and validation of a geriatric depression screening scale: a preliminary report. *J Psychiatr Res.* 1983;17:37-49.
13. Saracino RM, Weinberger MI, Roth AJ, Hurria A, Nelson CJ. Assessing depression in a geriatric cancer population. *Psycho-oncology.* 2017;26(10):1484-1490.
14. Burnam MA, Wells KB, Leake B, Landsverk J. Development of a brief screening instrument for detecting depressive disorders. *Med Care.* 1988;26(8):775-789.
15. Chang SC, Wang W, Pan A, Jones RN, Kawachi I, Okereke OI. Racial variation in depression risk factors and symptom trajectories among older women. *Am J Geriatr Psychiatry.* 2016;24(11):1051-1062.
16. Snowden M, Dhingra SS, Keyes CL, Anderson LA. Changes in mental well-being in the transition to late life: Findings from MIDUS I and II. *American journal of public health.* 2010;100(12):2385-2388.
17. Jones SM, LaCroix AZ, Li W, et al. Depression and quality of life before and after breast cancer diagnosis in older women from the Women's Health Initiative. *J Cancer Surviv.* 2015;9(4):620-629.
18. Ranchor AV, Wardle J, Steptoe A, Henselmans I, Ormel J, Sanderman R. The adaptive role of perceived control before and after cancer diagnosis: A prospective study. *Social science & medicine (1982).* 2010;70(11):1825-1831.
19. Trudel-Fitzgerald C, Savard J, Ivers H. Longitudinal changes in clusters of cancer patients over an 18-month period. *Health Psychol.* 2014;33(9):1012-1022.
20. Trudel-Fitzgerald C, Savard J, Ivers H. Evolution of cancer-related symptoms over an 18-month period. *J Pain Symptom Manage.* 2013;45(6):1007-1018.
21. Dunn J, Ng SK, Holland J, et al. Trajectories of psychological distress after colorectal cancer. *Psycho-oncology.* 2013;22(8):1759-1765.
22. Grimmett C, Bridgewater J, Steptoe A, Wardle J. Lifestyle and quality of life in colorectal cancer survivors. *Qual Life Res.* 2011;20(8):1237-1245.
23. Schlesinger S, Walter J, Hampe J, et al. Lifestyle factors and health-related quality of life in colorectal cancer survivors. *Cancer Causes Control.* 2014;25(1):99-110.

24. Loef M, Walach H. The combined effects of healthy lifestyle behaviors on all cause mortality: A systematic review and meta-analysis. *Prev Med.* 2012;55(3):163-170.
25. El-Shami K, Oeffinger KC, Erb NL, et al. American Cancer Society Colorectal Cancer Survivorship Care Guidelines. *CA: a cancer journal for clinicians.* 2015;65(6):428-455.
26. Kushi LH, Doyle C, McCullough M, et al. American Cancer Society Guidelines on nutrition and physical activity for cancer prevention: Reducing the risk of cancer with healthy food choices and physical activity. *CA: a cancer journal for clinicians.* 2012;62(1):30-67.
27. Trudel-Fitzgerald C, James P, Kim ES, Zevon ES, Grodstein F, Kubzansky LD. Prospective associations of happiness and optimism with lifestyle over up to two decades. *Prev Med.* 2019;126:105754.
28. Chasan-Taber S, Rimm EB, Stampfer MJ, et al. Reproducibility and validity of a self-administered physical activity questionnaire for male health professionals. *Epidemiology.* 1996;7(1):81-86.
29. Rimm EB, Stampfer MJ, Colditz GA, Chute CG, Litin LB, Willett WC. Validity of self-reported waist and hip circumferences in men and women. *Epidemiology.* 1990;1(6):466-473.
30. Rimm EB, Giovannucci EL, Stampfer MJ, Colditz GA, Litin LB, Willett WC. Reproducibility and validity of an expanded self-administered semiquantitative food frequency questionnaire among male health professionals. *Am J Epidemiol.* 1992;135(10):1114-1126; discussion 1127-1136.
31. Giovannucci E, Colditz G, Stampfer MJ, et al. The assessment of alcohol consumption by a simple self-administered questionnaire. *Am J Epidemiol.* 1991;133(8):810-817.
32. McCullough ML, Feskanich D, Stampfer MJ, et al. Diet quality and major chronic disease risk in men and women: moving toward improved dietary guidance. *Am J Clin Nutr.* 2002;76(6):1261-1271.
33. Stampfer MJ, Hu FB, Manson JE, Rimm EB, Willett WC. Primary prevention of coronary heart disease in women through diet and lifestyle. *N Engl J Med.* 2000;343(1):16-22.
34. Chiuve SE, Rexrode KM, Spiegelman D, Logroscino G, Manson JE, Rimm EB. Primary prevention of stroke by healthy lifestyle. *Circulation.* 2008;118(9):947-954.
35. Chiuve SE, Fung TT, Rimm EB, et al. Alternative dietary indices both strongly predict risk of chronic disease. *J Nutr.* 2012;142(6):1009-1018.
